# The Potential Role of Extracellular Vesicles in COVID-19 Treatment: Opportunity and Challenge

**DOI:** 10.3389/fmolb.2021.699929

**Published:** 2021-07-21

**Authors:** Yan-yan Yan, Wen-min Zhou, Yu-qing Wang, Qiao-ru Guo, Fu-xi Zhao, Zhuang-yan Zhu, Yan-xia Xing, Hai-yan Zhang, Mohamad Aljofan, Alireza Mosavi Jarrahi, Bolat Makabel, Jian-ye Zhang

**Affiliations:** ^1^ School of Medicine, Shanxi Datong University, Datong, China; ^2^ Key Laboratory of Molecular Target and Clinical Pharmacology and the State Key Laboratory of Respiratory Disease, School of Pharmaceutical Sciences and the Fifth Affiliated Hospital, Guangzhou Medical University, Guangzhou, China; ^3^ Xinjiang Institute of Materia Medica, Urumqi, China; ^4^ Department of Biomedical Sciences, School of Medicine, Nazarbayev University, Nur-Sultan, Kazakhstan; ^5^ School of Medicine, Shahid Beheshti University of Medical Sciences, Tehran, Iran

**Keywords:** COVID-19, SARS-CoV-2, extracellular vesicles (EVs), mesenchymal stem cell (MSC), immunomodulation, ACE2

## Abstract

SARS-CoV-2 infection has become an urgent public health concern worldwide, severely affecting our society and economy due to the long incubation time and high prevalence. People spare no effort on the rapid development of vaccine and treatment all over the world. Amongst the numerous ways of tackling this pandemic, some approaches using extracellular vesicles (EVs) are emerging. In this review, we summarize current prevalence and pathogenesis of COVID-19, involving the combination of SARS-CoV-2 and virus receptor ACE2, endothelial dysfunction and micro thrombosis, together with cytokine storm. We also discuss the ongoing EVs-based strategies for the treatment of COVID-19, including mesenchymal stem cell (MSC)-EVs, drug-EVs, vaccine-EVs, platelet-EVs, and others. This manuscript provides the foundation for the development of targeted drugs and vaccines for SARS-CoV-2 infections.

## Overview of COVID-19

### Current Prevalence of COVID-19

COVID-19, named Coronavirus Infectious Disease 2019 by the World Health Organization (Zhou, et al., 2020a; Zhu, et al., 2020), is an infectious disease caused by the severe acute respiratory syndrome coronavirus type 2 (SARS-CoV-2), which was first reported in Wuhan, China, in December 2019. COVID-19 infection is now a pressing global public health problem. As of today, 23 March 2021, more than 124 million people have been infected with SARS-CoV-2 and more than 2.7 million have died. In addition to having an unprecedented impact on global health care systems, COVID-19 has profound socioeconomic consequences (Nicola, et al., 2020).

Clinically, COVID-19 mainly affects the lungs. Although the majority of COVID-19 victims may show asymptomatic or mild symptoms, interstitial pneumonia (IP) and acute respiratory distress syndrome (ARDS) requiring mechanical ventilation in the intensive care units can occur in approximately 15% of cases (Cascella, et al., 2021), especially in the elderly and individuals with underlying diseases. COVID-19 also has systemic manifestations, affecting multiple organ systems containing cardiovascular, gastrointestinal, hematopoietic, renal, and immune systems (Yi, et al., 2020). In severe cases, COVID-19 can lead to severe cytokine storm or cytokine release syndrome (CRS) (Cascella, Rajnik, 2021), sepsis, multiple organ failure and even death.

SARS-CoV-2 is by far the seventh human coronavirus discovered to date. Four viruses (HCoV-NL63, HCoV-229E, HCoV-OC43, and HKU1) persist in human population and cause mild common cold symptoms (Fung,and Liu, 2019). The other two are similar to SARS-CoV-2, named SARS-CoV and Middle East Respiratory Syndrome (MERS)-CoV, and both of them lead to acute respiratory disease (Cui, et al., 2019). SARS-CoV-2 is a spherical or pleomorphic enveloped virus particle with a typical diameter range of 80–120 nm. This virus contains a 30 kB positive single-stranded RNA which surrounded by a membrane embedded with a variety of viral proteins, especially the Spike (S) protein (Mousavizadeh, and Ghasemi, 2020). Studies shown that the Spike protein in SARS-CoV-2 shared a high degree of structural homology with that in SARS-CoV (Li, et al., 2005; Xu, et al., 2020b). SARS-CoV-2 virus infect into human cells by recognizing the angiotensin converting enzyme 2 (ACE2) receptor of host cells. A recent study by Wan et al. reported that SARS-CoV-2 had a stronger binding ability with ACE2 than with SARS-CoV, promoting the infection and transmission capacity of the virus (Wan, et al., 2020).

### Pathogenesis of COVID-19

#### ACE2 Plays a Key Role in SARS-CoV-2 Invasion

In 2020, using cryopreserved electron microscopy, Zhou Qiang Laboratory of Westlake University successfully analyzed the full-length structure of ACE2 (Yan, et al., 2020), the receptor protein of SARS-CoV-2. This is the first time of the world that the full-length structure of ACE2 has been resolved. As SARS-CoV-2 invades the body, ACE2 acts like a “doorknob,” the virus grabs it and opens the door to the recipient cells. In addition, interestingly, it has recently been demonstrated that transmembrane protease serine 2 (TMPRSS2) incises the Spike protein during the internalization of SARS-CoV-2 which fuses with host cell membrane (Scheller, et al., 2019). This process is necessary for SARS-CoV-2 to enter into recipient cells (Devaux, et al., 2020). After fusing with the human cell membrane, the virus genome enters into the recipient/host cell. The virus then begins to replicate, mature and leave the host cells to infect new healthy cells. SARS-CoV-2 enters the respiratory system from the upper respiratory tract and eventually infects alveolar cells, causing angiectasis of alveolar cells, increased capillary permeability, decreased pulmonary surface active substances with infiltration of lymphocytes and monocytes. As ACE2 and TMPRSS2 are abundant in type II alveolar and endothelial cells (Hamming, et al., 2004), pulmonary vessels are susceptible to SARS-CoV-2-induced inflammation and injury (Zhao, et al., 2020). Other than the respiratory tract, SARS-CoV-2 infection can cause parts of the body damage, such as the cardiovascular system. Therefore, COVID-19 patients may present with severe forms of myocarditis and endocarditis besides respiratory dysfunction (Guzik, et al., 2020).

ACE2 is located in various types of epithelial cells (lung, kidney, heart, intestinal) and endothelial cells. In recent years, studies have shown that ACE2 played roles in the cardiovascular, renal and respiratory system, and was associated with hypertension and diabetes. ACE2 has a protective effect on a variety of lung diseases, such as acute lung injury, asthma, ARDS, pulmonary hypertension and chronic obstructive pulmonary disease (Jia, 2016). More importantly, ACE2 was identified as a SARS-CoV and SARS-CoV-2 receptor, which played a protective role in the pathogenesis of SARS and COVID-19. As the first homolog of ACE, ACE2 regulates the renin angiotensin system (RAS) by balancing the ACE activity. The binding complex of Spike protein with ACE2 can induce the cell membrane ACE2 down-regulation (Glowacka, et al., 2010), contributing to the imbalance of ACE and ACE2 activity, and therefore leading to acute lung injury (Zhang, et al., 2020a).

#### COVID-19 Is Associated With Endothelial Dysfunction and Micro-thrombosis

Patients with COVID-19 have a high incidence of thrombotic events (Berger, et al., 2020). About the mechanism of thrombosis in COVID-19, there are a lot of speculations, such as coagulation and platelet activation, endothelial cell activation, inflammation and complement system activation, etc (Mackman, et al., 2020). Varga and colleagues demonstrated that SARS-CoV-2 presented in the endothelial cells of various human organs (Varga, et al., 2020). In addition, evidence of alveolar capillary micro-thrombosis and endothelial injury associated with intracellular viruses has been noted in autopsy analyses of infected lungs (Ackermann, et al., 2020). Due to ACE2 show high-expression in vascular endothelial cells, viral infection of the circulatory system directly leads to excessive coagulation in COVID-19 patients (Varga, Flammer, 2020). In addition, the number of peripheral blood mononuclear cells decreased significantly in patients with respiratory failure 7–14 days after the onset of SARS-CoV-2 infection (Yi, Lagniton, 2020). High level of D-dimer was found to presence for the moment, which was associated with severe hypercoagulation and indicated a poor prognosis (Yi, Lagniton, 2020). As a major activator of the coagulation cascade (Grover, and Mackman, 2018), several studies have speculated that the induction of tissue factor (TF) might play an important role in the COVID-19-related thrombosis (Bautista-Vargas, et al., 2020; Grover and Mackman, 2018; Mackman, Antoniak, 2020). Axel Rosell et al. developed a method to determine the activity of TF in plasma EVs in 100 patients with moderate to severe COVID-19. The results showed that the level of EVs-TF activity was significantly higher in COVID-19 patients than in the normal individuals. In addition, the level of EVs-TF activity was associated with disease severity, mortality, and several plasma markers, including D-dimer. These findings suggest that SARS-CoV-2 infection induces the release of TF-positive EVs into the circulation, and possibly cause thrombosis in patients infected with COVID-19 (Rosell, et al., 2020).

#### Cytokine Storm: Immune Response to SARS-CoV-2 Is a Major Driving Force of Disease Severity

Because SARS-CoV-2 entry into cells depend on binding to its receptor, ACE2, the RAS and various inflammatory cascades are connected with the pathobiology of COVID-19 (Bourgonje, et al., 2020). The SARS-CoV-2 can also activate the innate and adaptive immune response in patients with COVID-19 (Yang, et al., 2020a). The immune effector cells release a large amount of proinflammatory cytokines and chemokines (Tukmechi, et al., 2014), such as tumor necrosis factor (TNF), interleukin 1 (IL-1), interleukin 6 (IL-6), interleukin 7 (IL-7) and granulocyte colony stimulating factor (Pedersen and Ho, 2020), inducing an uncontrolled CRS, and leading to various clinical manifestations, such as high fever, hepatosplenomegaly, cytopenia, central nervous system abnormalities, hypoalbuminaemia and capillary leakage (England, et al., 2021; Gao, et al., 2021). A previously published study showed that identifying circulating protein biomarkers in COVID-19 patients used an ultra-high-throughput serum and plasma proteomics technology (Messner, et al., 2020). Recently, Balaji Krishnamachary et al. indicated that EVs from patients affected by the SARS-CoV-2 might alter the pro-inflammatory response, blood coagulation disorders, and endothelium damage. They found that EVs of serious cases of COVID-19 carried higher levels of cytokines, including the IL-6 family, TNF superfamily, chemokines (MCP-1 and CXCL16), and proteases and peptidases (Cathepsin L1), compared with that of patients with moderate COVID-19 or asymptomatic individuals (Krishnamachary, et al., 2020). Therefore, the use of appropriate immunosuppressive and immunomodulatory agents to address the potential inflammatory complications of COVID-19 is currently being explored, which might improve clinical outcomes and ultimately reduce COVID-19 mortality (Stebbing, et al., 2020). Because ACE2 receptors are widely distributed in human alveolar type II cells and capillary endothelial cells (Hamming, Timens, 2004), the lungs are exceptionally sensitive to SARS-CoV-2 infection. In fact, recently published researches have shown that a substantial portion of the lungs were impacted by this disease, leading to an extensive damage that could subsequently result in permanent change of lung function. A recent MRI study in a 59-year-old man who diagnosed with COVID-19, suggested that this disease was not restricted to any specific area in the lung, but spread to the entire lung. In fact, this symptom is not composed directly arouse the SARS-CoV-2 virus, but a host immune response that causes a resistless cytokine storm in lungs. Overexpression of cytokines such as the interleukin family (IL-2, IL-6, IL-7), GSCF, IP10, MCP1, MIP1A, and TNF-α leads to edema and impairs oxygen exchange, which may lead to ARDS with potential acute cardiac injury, secondary infection and death (Huang, et al., 2020a).

## EVs Are the Promising New Therapeutic Means of COVID-19

There is currently no specific therapeutic for COVID-19. Conventional treatment includes infection prevention, supportive care that invloving supplement of oxygen and mechanical ventilation support (Grasselli, et al., 2020; Marini, and Gattinoni., 2020). Currently, drugs against COVID-19 are evaluated worldwide, including antiviral drugs, anti-malarial drugs and anti-inflammatory drugs, such as radecivir (Beigel, et al., 2020), chloroquine (Zhou, et al., 2020b) and hydroxychloroquine (HCQ) (Soy, et al., 2020), anthropoized anti-IL-6 receptor antibody tocilizumab (Perrone, et al., 2020; Toniati, et al., 2020; Xu, et al., 2020c), recombinant human IL-1 receptor antagonist Anakinra (Cavalli, et al., 2020; Huet, et al., 2020), etc. Although these treatment strategies improved patient recovery and survival, they do not definitively restore lung damage caused by the virus. In addition, a range of anti-inflammatory drugs have been tested to inhibit the cytokine storm and multiple organ failure caused by the worsening immune response in severe patients, but the effect has not been significant. In recent years, increasing researches reported that the role of EVs in the treatment of inflammation (Lasser, et al., 2016; Martinez-Bravo, et al., 2017), injury (Lanyu, and Feilong, 2019), and lung and respiratory viral infection (Scheller, Herold, 2019; van Dongen, et al., 2016; Yoshikawa, et al., 2019). It was reported that EVs promoted the pathogenesis of diseases such as in infectious diseases and cancers (Fleming, et al., 2014; Han, et al., 2019). The interesting interaction between EVs and the virus provides a new perspective on the treatment of COVID-19 (Dogrammatzis, et al., 2020). Viral infection may affect the exosomal-loading mechanisms of the host cells, resulting in changes in protein and nucleic acid content of EVs. It means the infected cells release modified EVs, not rely on virus contents. Therefore, compared with EVs without infected cells, these modified EVs may modulate the host immune response. Besides, EVs may act as a negatively regulatory element in the transmission of viral infection, and induce the immune system to respond to the virus. There are three types of EVs, including exosomes (20–150 nm), microvesicles (MVs) (100–1,000 nm in diameter), and apoptotic vesicles (1,000–5,000 nm) (Maione, et al., 2020; Raposo and Stoorvogel, 2013). EVs, as a carrier for cell-to-cell transfer of biomolecules, is an important mode of cell-to-cell communication (Huang, et al., 2020b). EVs are present in a variety of biological fluids, such as blood, tissue fluid, pleural fluid, bronchoalveolar lavage fluid (BAL), peritoneal fluid, saliva, urine, breast milk, cerebrospinal fluid, amniotic fluid and so on (Kowal, et al., 2014; Rezaie, et al., 2019). EVs can be transported or accumulated not only in biological liquids but also in solid tissues. In solid tissue, EVs deliver their contents, such as proteins, miRNAs, mRNAs, and lncRNAs, to adjacent or distant cells and reprogram the target cells in fate, function, and morphology, resulting in physiological or pathological effects (Kowal, Tkach, 2014; Maas, et al., 2017; Statello, et al., 2018).

Exosomes, also referred to as intraluminal vesicles (ILVs), are a subtype of EV formed by an endosomal route, which are surrounded by a phospholipid bilayer, and have been found in biological liquids (Raposo and Stoorvogel, 2013). Exosomal vesicles form through inward budding of the limiting membrane of early endosomes, which mature into multivesicular bodies (MVBs) (Huang, Yan, 2020b). MVBs play a significant role in the endocytic and trafficking functions of the cell material, such as protein sorting, recycling, storage, transport, and release (Babaei, and Rezaie, 2021). Release of exosomes into the extracellular space is facilitated by the fusion of the MVB limiting membrane with the plasma membrane. Researches showed that exosomes participated in cell communication, cell maintenance, and tumor progression (Rezaie, et al., 2021). Balaji Krishnamachary et al. explored EVs isolated from plasma of patients with COVID-19 to identify some potential biomarkers which influence the disease severity and to analyze its role in the pathogenesis of this disease (Krishnamachary, Cook, 2020). Plasma-derived EVs were separated from 53 COVID-19 patients in hospital and compared depending on the severity of their disease. Analysis of inflammation and cardiovascular protein loading in large EVs suggested significant differences in protein expression across disease subgroups. Prominently, the TNF superfamily and IL-6 family members were upregulated in severe and moderate disease patients who need oxygen supplement. EVs in severe patients also shown enhancement of prothrombotic or endothelial injury factors (TF, t-PA, and VWF) and cardiovascular pathology-related proteins (MB, PRSS8, REN, and HGF). There were significantly higher levels of TF, CD163, and EN-RAGE has been observed in EVs from patients with severe disease compared to moderate disease. EVs also play a key role in transmitting viral infection (Owczarek, et al., 2018). It was found that EVs and viruses shared similar physicochemical properties. They are small in size and have common biogenesis and cell entry mechanisms (van Dongen, Masoumi, 2016). The virus enters the uninfected cell *via* the endocytosis pathway and then exits the host cell by budding directly through the cell membrane. A new study found that EVs acted as the tool of virus export to cell, and the EVs depend virus entry mechanisms for cargo transport. EVs are the delivery vectors of viral contents. EVs released from virus-infected cells can infect healthy cells by transferring viral components, for example, virus-derived miRNAs and viral proteins, etc. (Ali, et al., 2010; Arenaccio, et al., 2015; Nolte-'t Hoen, et al., 2016). EVs separated from infected cells may also activate humoral and cellular immune responses by transferring viruses and autoantigens in the host (Fleming, Sampey, 2014; Gunasekaran, et al., 2017). In addition, EVs release from virus-infected cells can also transfer viral receptors and pro-inflammatory factors to the recipient cells, leading to the transmitting of viral infection and worsening of tissue damage (Mack, et al., 2000). Studies have shown that the number of EVs secreted from the SARS-CoV-2 infected cells increased significantly during the virus infection, and EVs still played an important role in the pathogenesis of diseases (Yoshikawa, Teixeira, 2019). Lanyu et al. emphasized the role of EVs play which secreted by lung cells and alveolar epithelial cells in lung injury and inflammation (Lanyu and Feilong, 2019). According to report, EVs secreted from broncho alveolar lavage fluid are involved in the pathogenesis of idiopathic pulmonary fibrosis through the signaling regulation mediators such as Wnt5a (Martin-Medina, et al., 2018). The latest research showed EVs derived from epithelial cells which transduced by lentiviral overexpressing SARS-CoV-2 gene could transfer viral genes to recipient cardiomyocytes, leading to increased expression of inflammatory genes (Kwon, et al., 2020).

**FIGURE 1:**
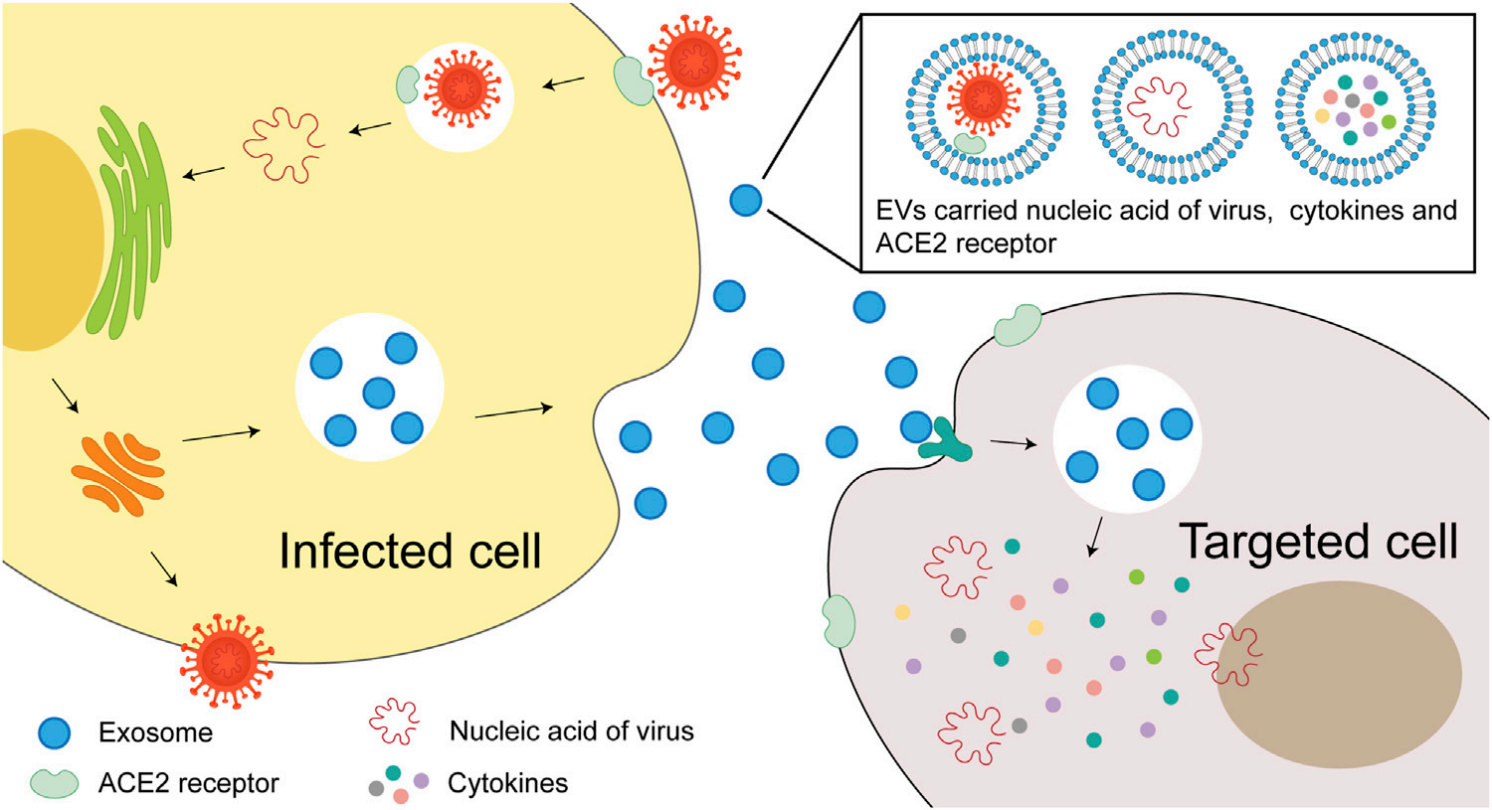
The role of EVs in SARS-CoV-2 virus internalization and infection. EVs may contribute to the transmission of SARS-CoV-2 virus. The virus may enter the recipient cells through internalization. Virus components, such as miRNAs, viral proteins and viral receptor ACE2, are packaged into the EVs and make the healthy cells sensitive to viral invasion.

EVs may contribute to the infection, internalization and transmission of SARS-CoV-2 virus. Some components such as miRNAs, viral proteins and viral receptor ACE2 could be packed into EVs (Wang, et al., 2020), that render the recipient cells sensitive to viral invasion (Figure 1). In this review, we discuss the current EVs based COVID-19 treatment strategies, including MSC and its EVs, EVs based drug delivery systems, EVs based vaccine, platelet EVs and inhibition of EVs intake, etc.

## EVs-based COVID-19 Therapy

### MSC and MSC-EVs

MSCs are heterogeneous cells, including stromal cells, progenitor cells, fibroblasts, and stem cells (Dominici, et al., 2006; Galderisi and Giordano, 2014). The MSCs can be isolated from many tissues, such as bone marrow, placenta, adipose tissue, and umbilical cord blood. They are currently used as therapeutics at the present time, and meanwhile being tested in multiple clinical trials across the world (Meng, et al., 2020). MSCs are safe and possess immunomodulatory and tissue regeneration capabilities (Gao, et al., 2016; Thompson, et al., 2020). Studies suggested that the prospective therapeutic products based on MSCs were success, despite the apparent heterogeneity origin and lacking in specific biomarkers to predict, once implanted, MSCs could show strong regulate ability in immunomodulatory, antioxidant, and angiogenesis. MSCs and MSC-EVs have emerged as promising novel therapies that can not only reduce inflammation, but also regenerate and repair lung damage, and may therefore be used alone or in combination with other therapeutic agents to benefit patients with COVID-19.

Due to MSCs lack the expression of membrane bound molecules involved in immune rejection which enable their allogenic transplantation, the clinical applications of MSC-based therapy have witnessed an outstanding achievement (Ahmadi and Rezaie, 2021). Nevertheless, in the long-term follow up, safety issues regarding MSCs-based therapy are still a matter of debate. It is worth noting that MSCs have the capacity to differentiate into endothelial cells and to create a capillary network, and the inhibitory effect of MSCs in anti-tumor immune response results an increased tumor growth, which promotes tumor growth and metastasis (Kolios and Moodley, 2013). In addition, it was reported that local microenvironment in which MSCs engraft contained factors that induced unwanted differentiation of transplanted MSCs *in vivo*. Therefore, several factors and signaling pathways regarding MSCs therapy after their *in vivo* administration should be focused. Medical staff should abide by moral and ethical norms, so that the public and government administrators can clearly comprehend the nature and functions of MSCs, and understand the potential risks of treatment, and avoid deviating by non-standard clinical trials (Volarevic, et al., 2018).

The membrane of MSC-EVs is rich in cholesterol, sphingomyelin, ceramide and lipid raft proteins, which enables membrane fusion with target cells. After fusion, MSC-EVs may trigger signaling pathways through the receptor ligand interaction, or be internalized by endocytosis to deliver contents, such as mRNAs, miRNAs, enzymes, cytokines, etc. (Harrell, et al., 2019). Compared with homocellular transport, MSC-EVs overcome safety concerns regarding the long-term survival of engrafted MSCs due to its composition (Rezaie, et al., 2018). Thus, MSC-EVs therapy is a new remedy in cell-free treatment of autoimmune and inflammatory diseases.

#### Application of MSCs in Pulmonary Diseases

**FIGURE 2:**
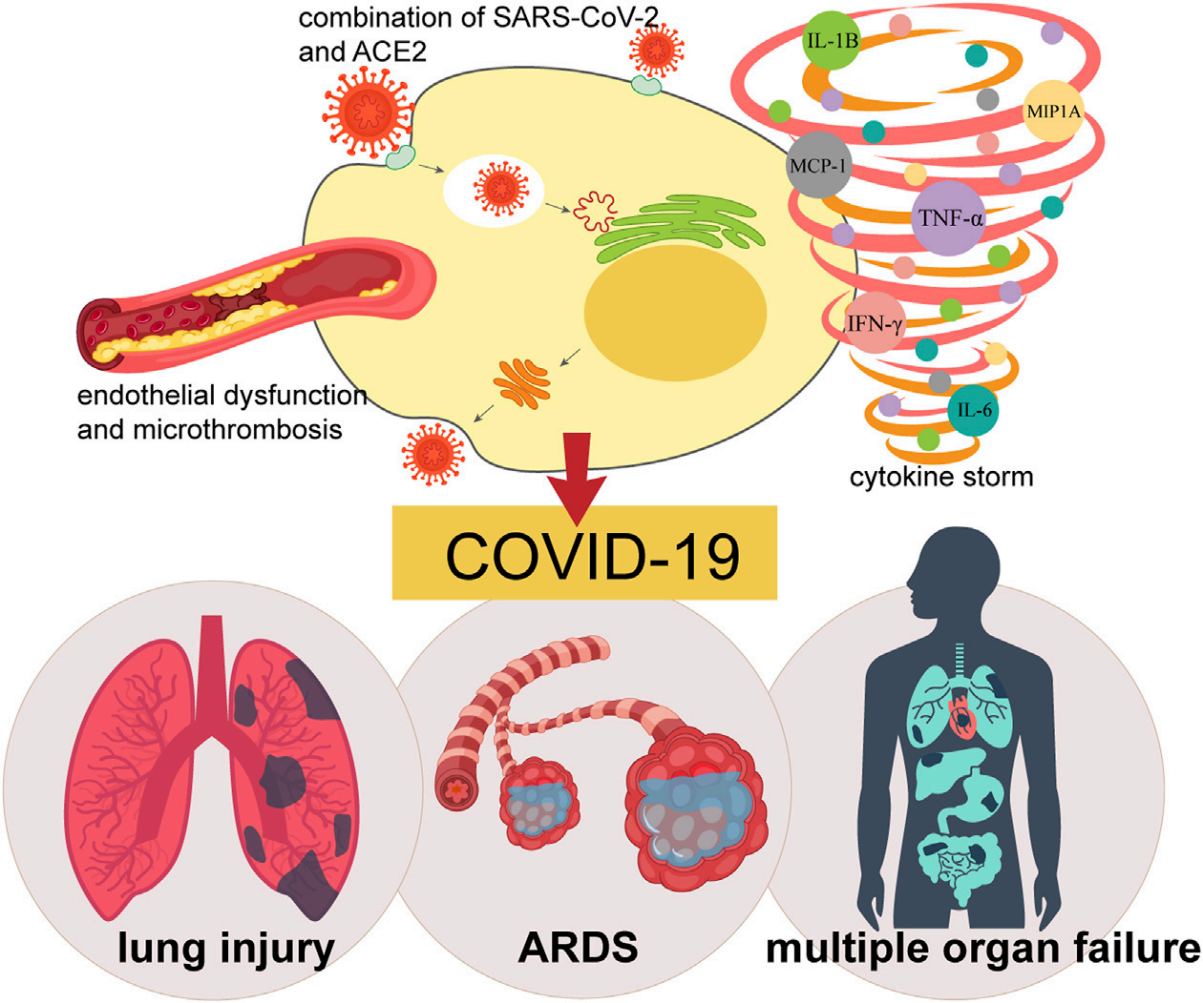
Pathogenesis of COVID-19. The combination of SARS-CoV-2 and virus receptor ACE2, endothelial dysfunction and micro thrombosis, together with cytokine storm leads to COVID-19, causing a series of clinical manifestations such as lung injury, ARDS, and even systemic multiple organ failure.

Inflammatory cytokines play a leading role in the development of COVID-19-caused lung injury (Figure 2). Immunotherapy that reduce the cytokine storm could be the key treatment option. However, traditional immunotherapy usually targeted one or two factors and may not produce enough response. MSCs have been shown to have powerful and extensive immunomodulatory and anti-inflammatory capabilities (Abdi, et al., 2008; Wada, et al., 2000).

Bone marrow MSCs therapy has evolved from preclinical trials to clinical trials for different diseases states in recent years. In animal models, MSCs significantly improve lung pathological change (Cruz and Rocco, 2020) and inhibit immune cell-mediated inflammation induced by influenza virus (Khatri, et al., 2018). Bhattacharya et al. demonstrated that bone marrow MSCs could stabilize endothelial cells and maintain alveolar-capillary barrier function, which is essential for maintaining or reducing inflammation-induced lung permeability, thereby mitigating the development of interstitial pulmonary edema (Bhattacharya and Matthay, 2013). Li et al. reported that MSCs could alleviate acute lung injury caused by H9N2 and H5N1 viruses in mice by reducing the secretion of pro-inflammatory chemokines and cytokines, and inhibiting migration of inflammatory cells to the lungs (Li, et al., 2016). Currently, there are several ongoing clinical trials using MSCs for a variety of pulmonary diseases, such as obstructive bronchiolitis idiopathic pulmonary fibrosis (NCT02013700), chronic obstructive pulmonary disease (NCT01849159), and bronchial dysplasia (NCT01175655) (Wecht and Rojas, 2016), as well as for ARDS (NCT02097641) (Matthay, et al., 2019) and septic shock (NCT02421484) (McIntyre, et al., 2018). Specifically, Leng et al. found that there were no ACE2 and TMPRSS2 receptors express in MSCs, which indicated that the virus should not infect this cell population (Leng, et al., 2020). In a rat model of hyperoxia-induced lung injury, MSCs reduced overexpression of hyperoxia-induced angiotensin and angiotensin type 1 receptor (AT1R), and reduced ACE to normal level (Chen and Chou, 2018). Recently, Simonson et al. reported a long-term follow-up study on two severe ARDS patients, who required ECMO support and combined mechanical ventilation during the acute phase (Simonson, et al., 2020). After receiving a single systemic infusion of allogeneic MSCs, both patients fully recovered their physical and mental abilities. Remarkably, a dual-energy CT scan showed no signs of pulmonary fibrosis after 5 yr of MSCs treatment.

#### Application of MSCs in the Treatment of COVID-19

MSCs therapy is considered a promising strategy for the treatment of COVID-19. The potential of MSCs for the treatment of COVID-19 is based on two benefits: *1*) induction of the regenerative program of lung epithelial and endothelial cells, and *2*) synchronous modulation of the inflammatory response. MSCs secrete multiple kinds of cytokines and paracrine factors which interact with immune cells directly, including T cells, B cells, dendritic cells, macrophages, and natural killer cells. This combined effect enables MSCs have immunomodulatory capability, which contributes to suppressing overactivation of the immune system. By producing and recruiting different growth factors such as vascular endothelial growth factor (VEGF), epidermal growth factor (EGF) and transforming growth factor (TGF), MSCs promote tissue regeneration and thus improve the microenvironment (Atluri, et al., 2020). It prevents uncontrolled inflammatory cascades while reducing pulmonary fibrosis and pulmonary dysfunction following COVID-19 infection (Mo, et al., 2020; Sun, et al., 2020; Thille, et al., 2013). Particularly, the lacking of ACE2 ensures the injected MSCs achieve immunomodulatory effects free from being destroyed by virus. MSCs from human umbilical cord origin were reported in a recent clinical trial, compared to the placebo group, they were associated with the increase of peripheral lymphocyte count and the decrease of systemic inflammatory biomarkers (ChiCTR2000029990) (Leng, et al., 2020). MSC infusion alleviated cytokine storm syndrome and significantly improved outcomes in patients with severe COVID-19.

#### Application of MSC-EVs in Lung Diseases

Most of the therapeutic progress of MSCs are achieved through paracrine mechanisms, including EVs secretion that contain cell protective factors such as keratinocyte growth factor (KGF), anti-inflammatory mediators (PGE2 or lipid A4), anti-osmotic factors (Ang1) and others (Doorn, et al., 2012; Gnecchi, et al., 2008; Li, et al., 2014; Silini, et al., 2017). There have been reports of some limitations of using MSCs. For instance, intravenous administration of MSCs may induce particle aggregation leading to embolism; and some MSCs, especially those from embryonic tissues, may carry a risk of mutagenicity and tumorigenicity. Therefore, MSC derivatives such as SECRETOME (Bari, et al., 2019) and EVs (Tsiapalis and O’Driscoll, 2020) have been suggested as alternatives to MSC therapy. In addition, account of the low immunogenicity and tumorigenicity, simplicity of operation, and low cost, MSC-EVs therapy has significant advantages over MSC therapy. According to the report recently published, MSC-EVs could be administered by inhalation or injection (Bari, Ferrarotti, 2019). Preliminary studies have shown that MSC-EVs might also be effective against COVID-19 (Bari, et al., 2020). MSC-derived EVs have been demonstrated to induce similar influence to parent cells. In animal models, they can be store for a longer time safely without losing their biological function, on the other hand, they have been indicated similar or better indications than MSCs (Tsiapalis and O'Driscoll, 2020).

Some researchers have demonstrated the immunomodulatory effects of MSC-derived EVs *in vitro* (Budoni, et al., 2013; Del Fattore, et al., 2015; Di Trapani, et al., 2016; Henao Agudelo, et al., 2017; Liu, et al., 2012), and have shown significant anti-inflammatory and regenerative abilities in several diseased animal models (Fierabracci, et al., 2015; Phinney and Pittenger, 2017). Specifically, researchers showed demonstration that in animal models of lung injury, the efficacy of MSC-EVs was effective like in hyperoxia (Braun, et al., 2018; Porzionato, et al., 2019; Willis, et al., 2018), severe bacterial pneumonia (Monsel, et al., 2015), and viral pneumonia (Khatri, Richardson, 2018). In addition, MSC-EVs has beneficial effects on lung perfusion *ex vivo* with severe *E. coli* pneumonia (Park, et al., 2011). Finally, MSC-EVs can effectively repair marginal donor lungs through several methods, the first one is dose-dependently increasing the clearance of alveolar fluid; the second one is reducing lung weight after perfusion and ventilation; and the third one is improving airway and hemodynamic parameters (Ragni, et al., 2017). On the basic of these useful and promising results, the role of MSC-EVs in alleviating and repairing ARDS lung injury is gaining increasing attention (Lee, et al., 2019; Li, Huang, 2014; Monsel, Zhu, 2015; Shah, et al., 2019). Notably, fibrosis sequelae with reduced pulmonary function have been reported in patients recovering from COVID-19 pneumonia. Indeed, there is an increased risk of idiopathic pulmonary fibrosis following viral infection (Sheng, et al., 2020), as a long-term complication, it has been reported in parts of SARS infected patients (Zhang, et al., 2020b). MSC-EVs prevent fibrosis following experimental lung injury, which is similar to the cells of their origin (Cargnoni, et al., 2009; Mansouri, et al., 2019). In fact, there is a growing interest in the new potential application of EV as a disease treatment strategy. EVs are considered safe, easy and cheap to produce, isolate, store and manage (Muraca, et al., 2017), which could reduce costs and improve product availability. It is worth mentioning that EVs seem to be a very generic product that it has application diversity and can be modified by various technologies, for example, manipulating their parent cells use genetic engineering, and then integrated into the secreted EVs by introducing exogenous substances (Armstrong, et al., 2017; Vader, et al., 2016; Wiklander, et al., 2019). A similar method has been demonstrated to deliver exogenous miRNA-Let-7c to alleviate renal fibrosis *via* MSC-EVs in the mice model of unilateral ureteral obstruction (Wang, et al., 2016). On the other hand, studies also suggested that EVs could be loaded with therapeutic molecules form a polymer complex to improve its targeting activity (Cappariello, et al., 2018; Pascucci, et al., 2014; Vader, Mol, 2016; van der Meel, et al., 2014).

The pulmonary pathology of COVID-19 critically ill patients includes the exudation and proliferation phase of diffuse alveolar injury, and microvascular thrombosis that suggest early ARDS (Wichmann, et al., 2020; Xu, et al., 2020d). The pathogenesis includes change of alveolar permeability and neutrophils infiltration (Zemans and Matthay, 2017). Several studies showed that MSC-EVs could reduce alveolar permeability and increase alveolar fluid clearance, in an vitro model of human perfusion lung injury with severe *E. coli* pneumonia (Gennai, et al., 2015; Lee, et al., 2009; Park, et al., 2019). Interestingly, there were antibacterial substances in the culture medium of the bacteria-stimulated MSCs (Krasnodembskaya, et al., 2010). In a mouse sepsis model, treatment with MSCs could increase bacterial clearance, part of the reason is it increased phagocytic activity of host immune cells (Mei, et al., 2010). In addition, MSC-EVs demonstrated antiviral activity *in vitro* by inhibiting replication of influenza virus in lung epithelial cells, meanwhile by reducing viral load in an influenza-induced lung injury model of pigs (Khatri, Richardson, 2018).

#### Application of MSC-EVs in the Treatment of COVID-19

Recently, Sengupta, et al. published the safety and efficacy of allogeneic bone marrow MSC-derived EVs in 24 patients with COVID-19 severe pneumonia that they have been treated with hydroxychloroquine and azithromycin (Sengupta, et al., 2020). It was interesting to note that inflammatory biomarkers and absolute neutrophilic counts were decreased prominently, while the counts of total lymphocyte and CD8^+^ were increased prominently within 5 days after EVs injection. In addition, D-dimer was significantly reduced. Although this EVs treatment resulted about 71% of patients recovery to health, it is not clear whether the recovery was attributable to EVs treatment, because of the lack of a matched control group and the lack of reported protein or microRNA composition of EVs used in the study. In summary, we would like to underline that there has been currently no approved MSC-based approach in preventing and treating the COVID-19, either with MSCs or MSCs-derived EVs. Clinical trials using EVs should be carried out with great caution, as their cargo determines the functional impact. Consequently, before MSC-derived EVs used in clinic, standardized protocols need to be developed, including mass production, isolation, functional evaluation, and batch-to-batch consistency. Some clinical trials using MSC-EVs in COVID-19 is currently underway and are summarized in Table 1.

**TABLE 1:** Ongoing clinical trials of MSC-EVs in COVID-19.

Registration number	Study title	Number enrolled	Interventions	Purposes
NCT04276987	A pilot clinical study on inhalation of mesenchymal stem cells exosomes treating severe novel coronavirus pneumonia	24	MSCs-derived exosomes	To explore the safety and efficiency of aerosol inhalation of the exosomes derived from allogenic adipose mesenchymal stem cells (MSCs-Exo)
NCT04798716	The use of exosomes for the treatment of acute respiratory distress syndrome or novel coronavirus pneumonia caused by COVID-19	55	MSC-exosomes delivered intravenously	To explore the safety and efficacy of an intravenous injection of MSC derived exosomes
NCT04491240	Evaluation of safety and efficiency of method of exosome inhalation in SARS-CoV-2 associated pneumonia. (COVID-19EXO)	30	EXO inhalation	To explore the safety and efficiency of aerosol inhalation of the exosomes
ChiCTR2000030261	A study for the key technology of mesenchymal stem cells exosomes atomization in the treatment of novel coronavirus pneumonia (COVID-19)	26	Aerosol inhalation of exosomes	To inhibit inflammatory factors and enhance the immunity of the body, promoting the early recovery of patients and reducing complications
ChiCTR2000030484	HUMSCs and exosomes treating patients with lung injury following novel coronavirus pneumonia (COVID-19)	90	Intravenous infusion of HUMSCs and exosomes	To evaluate the safety and efficacy of the treatment of human umbilical cord mesenchymal stem cells (MSCs)

Detailed information can be searched at https://clinicaltrials.gov and http://www.chictr.org.cn.

Preliminary studies demonstrated that intravenous injection of MSCs and MSC-EVs possessed an enormous potential in treatment for COVID-19 patients. So far, no adverse events were found during MSCs and MSC-EVs treatment and follow-up period, suggesting that this therapy is safe and effective for COVID-19 patients (Akbari and Rezaie, 2020). However, MSCs express tissue factors and accumulate mainly in pulmonary capillaries, which may increase the risk of pulmonary embolism and other thromboembolic events. The effects of administration time, dose, frequency and route of MSCs and MSC-EVs need further study. Though clinical outcomes are promising, the limited literature still warrants more studies to establish safety and efficacy of MSCs and MSC-EVs to treat and manage symptoms associated with COVID-19 infection (Hamdan, et al., 2021).

### Drug-EVs

One interesting approach of using EVs as therapeutic agents is its drug delivery potential (Gnecchi, et al., 2006; Lamichhane, et al., 2016; Lv, et al., 2012). EVs have a cellular origin, such as MSCs, and therefore offer greater safety and stability than other delivery systems such as liposomes (Malhotra, et al., 2016). Selectable compounds or genetically engineered molecules may loading with EVs in the nanocarriers to enhance the targeting ability for tissue/cell infection, suggesting that EVs-based nanocarriers can be used to treat infectious diseases. Some studies have used EV as an effective carrier of tumor-targeted anticancer drugs curcumin, doxorubicin, and paclitaxel (Kim, et al., 2016). Therefore, an EV-based drug delivery system has great potential to increase drug loading in targeting cells and inhibit off-target effects. EVs may be used to deliver therapeutic drugs or biomodulators to inhibiting the spread and replication of virus in the recipient cells (Romagnoli, et al., 2014). The development of safe and efficient nanocarriers is the main goal of nanomedicine. Therefore, the development of EVs-based nanocarriers offers a promising opportunity for therapeutic drug delivery. However, most of the studies have been conducted *in vitro* and *in vivo* experimental models, but it remains a mystery of the safety, specificity, and efficiency of the method in clinical trials.

Another attractive treatment option is to use the blood products of convalescent patients, such as the whole blood, plasma or serum. The EVs containing neutralizing antibodies in convalescent blood products were transferred into patients, and we benefit tremendously with this treatment by promoting immune regulation and lung tissue wound healing. Kesimer et al. proved that EVs derived from culture medium of human tracheobronchial epithelial cells showed a neutralizing effect on human influenza virus (Kesimer, et al., 2009). Studies have proved that plasma-derived EVs also carry an amount of cell growth factors, and these factors could induce the activation of cellular signaling pathways, change vascular reactivity, induce angiogenesis and promote tissue repair (Guo, et al., 2017; Tao, et al., 2017; Torreggiani, et al., 2014).

### EVs Vaccines

The use of EVs as immunogenicity factors in the treatment of SARS coronavirus infection has been studied. EV might be used as a vaccine with its advantages of high stability, low toxicity and immunogenicity in circulation (Zhou, et al., 2020c). Kuate et al. analyzed exosome-based vaccines which containing the S protein of SARS-CoV-2. The S proteins-containing exosomes were procured by replacing the transmembrane and cytoplasmic domains of the S protein with those of VSV-G, and its immunogenicity and efficacy were tested in mice. After comparing it to an adenoviral vector vaccine expressing the S protein, it has been proved that both of the exosomes and the vaccine could induce neutralizing antibody titers. After priming with the SARS-S protein exosomal vaccine and boosting with the adenoviral vector the neutralizing antibody titers of using SARS-S protein exosomal vaccine exceeded those observed in the convalescent serum of SARS patients (Kuate, et al., 2007). In addition, the treatment of EVs has been shown to be more effective than that of soluble protein subunit vaccines, which might be due to the fact that the expression of multiple copies of the same viral protein exposed on the EVs surface promotes the cross-linking between EVs and the B cell receptor (Gyorgy, et al., 2015).

### Platelet-derived EVs

Platelets are small (2–4 µm), and they are the anuclear cellular fragments of megakaryocytes from bone marrow and lung (Lefrancais, et al., 2017; Machlus, and Italiano, 2013). There are almost one trillion platelets patrol in the blood to hold the vascular system integrity. Once blood vessels are damaged, platelets will form a thrombus to prevent subsequent hemorrhage (Davi and Patrono, 2007). Platelet mediated thrombosis may also involve EVs (micro particles or micro vesicles), that provide anionic phospholipids such as phosphatidylserine to support the blood coagulation cascade (Ridger, et al., 2017). In addition to playing a role in hemostasis and thrombosis, platelets contribute to inducing the immune and inflammatory response (Kapur, et al., 2015; Li, et al., 2012; Morrell, et al., 2014; Semple, et al., 2011). The exosmosis and invasion to inflammatory tissues of neutrophils require interaction with activated platelets (Imhof, et al., 2016; Sreeramkumar, et al., 2014). The release of extracellular DNA (NETosis) by neutrophils was observed in patients with COVID-19 (Barnes, et al., 2020; Middleton, et al., 2020; Zuo, et al., 2020). NETosis requires platelets and may result in thrombosis (Constantinescu-Bercu, et al., 2020; Martinod, and Wagner, 2014). Some immune and inflammatory molecules have been found to express in platelets, such as IL-1; besides some immune receptors have been detected, such as CD40L, Toll-like receptors (TLR), and Fc receptors (Karas, et al., 1982; Lindemann, et al., 2001; Semple, Italiano, 2011).

Serological findings in patients with symptomatic COVID-19 include severe leukopenia and lymphocytopenia (Arentz, et al., 2020; Connors and Levy, 2020; Tang, et al., 2020). Low platelet count is closely associated with an increased risk of death for the in-hospital COVID-19 patients, although platelet levels are not generally considered clinically relevant. This finding suggests that SARS-CoV-2 infection may decrease platelet production and/or increase the damage of platelet. Patients with COVID-19 are more likely increase platelet consumption because of the platelet activation and thrombosis (Al-Samkari, et al., 2020; Liao, et al., 2020; Lippi and Favalora, 2020a; Lippi, et al., 2020b; Liu, et al., 2020; Xu, et al., 2020a; Yang, et al., 2020b).

Since thrombus and clotting are mainly controlled by platelets (Roberts, et al., 2006), it is critical to determine the status of platelets in COVID-19. Platelet-EVs delivers molecules from the mother platelet. They transport platelet-derived cytokines and other pro-inflammatory molecules, such as damage-associated molecular patterns (DAMPs) (Boudreau, et al., 2014; Maugeri, et al., 2018; Melki, et al., 2017). SARS-CoV-2 enters the ACE2-expressed endothelial cells. The loss of endothelial integrity may facilitate the recruitment of circulating platelets to the infected site, causing platelet activation and degranulation. Through analysis of platelet degranulation and cytokine release, Zaid et al. observed an evident increase in platelet-EVs firstly in platelet-EVs in patients with COVID-19 (Zaid, et al., 2020). This increase may be due to increased production of megakaryocytes or platelets, reduced clearance, or a combination of the two. Surprisingly, the level of platelet-EVs was significantly lower in severely ill patients than in non-critically ill patients. In severe COVID-19 cases, the number of platelets decreases. They reported that platelets were at the forefront of COVID-19 because they release various molecules at different stages of the disease. Thus, it is possible that platelets, which are associated with SARS-CoV-2 RNA and are highly activated in COVID-19, may be involved in the overwhelming thrombosis of COVID-19. Platelet overactivation may be involved in the systemic inflammatory response and thrombosis events observed in this disease. Therefore, inhibition of pathways associated with platelet activation may improve prognosis during COVID-19.

Giuseppe Cappellano et al. (Cappellano, et al., 2021) showed that platelet-EVs counts were higher in SARS-CoV-2 positive patients compared with SARS-CoV-2-negative patients, while platelet counts were not changed. What is particularly interesting is that the level of PLT-EVs was strongly associated with SARS-CoV-2 infection by a multivariate analysis, which independent from any confounding factors (age, sex, comorbidity, etc.). There was a very good diagnostic performance in ROC curve analysis, with sensitivity of 75% and specificity of 74%.

Platelet-derived EVs can be used to develop novel treatment strategies for COVID-19 patients. A study showed that engineered platelet-derived EVs loaded with TPCA-1 were effective in treating pneumonia (Ma, et al., 2020). In mouse models that selectively target inflammatory sites, platelet-derived EVs suppressed inflammation and reduced local cytokine storms.

### Reduces EVs Secreted by Virus-infected Cells Into Receptor Cells

EVs secreted from virus-infected cells contribute to promoting viral infection and inhibiting immune cell response. Therefore, it may be a new useful method to overcome viral transmission by inhibiting the uptake of EVs (Li, et al., 2019; Schneider, et al., 2017).

## Opportunities and Challenges

Understanding the roles of EVs in COVID-19 infection could increase our knowledge of the dynamics of the virus and help developing effective prevention and treatment modalities. The above-mentioned findings provide a basis for future research on the dynamics of SARS-CoV-2 virus infection and its inhibition. However, it is worth noting that no EVs-based treatment has been approved to date. Significant barriers remain for the development of MSC-EVs as a therapeutic tool (Tsiapalis and O'Driscoll, 2020). Currently, we do not have any information on the possible side effects of EVs treatment against COVID-19. First, there are the similarities between EVs and viruses, both of them interact with the endosomal system which is responsible for synthesis of EVs. Some viruses may use this mechanism to pack viral proteins and RNA into vesicles in infected cells, subsequently released into the extracellular space, then facilitate the spread of the virus to uninfected cells (Gould, et al., 2003). This process has been demonstrated in HIV infection, but has not been elucidated in coronavirus infection (Arenaccio, Anticoli, 2015; Giannessi, et al., 2020). Second, the heterogeneity of EVs is a big challenge (Tkach, et al., 2018). Up to now, all published studies using heterogeneous EVs in some cases using the part size (<200 nm “small” EVs). Because the origin, size, composition and functional characteristics of the EVs are different, the source selection should be carefully described when using them for treatment. It is important to note that different sizes EVs of dendritic cells can induce the activation of T cells with different polarization mode (Kowal, et al., 2016). Third, due to the lack of standardization for accurate counting EV, as well as the current equipment does not differentiate between vesicles and non-vesicular granules, the quantitation of EV preparation is also an unsolved problems (Tkach, Kowal, 2018). For example, Campanella et al. reported that autologous exosomes were safer than allogeneic plasma exosomes in regenerative medicine (Campanella, et al., 2019). Soni et al. demonstrated that alveolar macrophages produce EVs might have a proinflammatory or anti-inflammatory effects, depending on the separation time of EVs in the process of acute lung injury (Soni, et al., 2016). Fourth, variability of tissue of origin and culture conditions (Patel, et al., 2018; Pittenger, et al., 2019). Although the MSCs from different sources have different immunosuppression and differentiation ability, but the optimal source of immunomodulation has not yet been determined (Gao, Chiu, 2016). Chance et al. reported that MSC-EVs had higher thrombotic activity than BMMMSC-EVs (Chance, et al., 2019). Recently, Inal reported a specific EVs subgroup, especially the EVs-TF positive might be associated with venous thromboembolism in COVID-19 patients with hypertension and diabetes (Inal, 2020). Taken together, it is critical to emphasize that care should be taken in using EVs for disease treatment (Borger, et al., 2020). To ensure the curative effect and safety of using EVs for COVID-19, enormous efforts are required to improve the technology for the separation and identification of EVs.
